# Short-read genome skimming enables molecular barcoding of old myxomycete collections

**DOI:** 10.3897/imafungus.17.201932

**Published:** 2026-09-03

**Authors:** Dmytro V. Leontyev, Martin Schnittler, Oleg N. Shchepin

**Affiliations:** 1 H.S. Skovoroda Kharkiv National Pedagogical University, Alchevskikh 29, Kharkiv 61002, Ukraine H.S. Skovoroda Kharkiv National Pedagogical University Kharkiv Ukraine; 2 Institute of Botany and Landscape Ecology, University Greifswald, Soldmannstr. 15, Greifswald D-17487, Germany Institute of Botany and Landscape Ecology, University Greifswald Greifswald Germany

**Keywords:** *
Amoebozoa
*, bioinformatics, DNA degradation, herbarium genomics, metagenomics, next-generation sequencing, nucSSU, old DNA

## Abstract

This study evaluates the effectiveness of Illumina-based genome skimming for barcoding myxomycete herbarium collections ranging from 29 to 91 years in age. We successfully retrieved partial sequences of the standard marker gene (nucSSU) in all cases, as well as additional markers (mtSSU, EF1a, and COI) for certain collections. Altogether, 28 genes were recognized in the studied material. In a 33-year-old specimen of *Lindbladia tubulina*, the assembly reached an N50 of 4.19 kb, enabling the recovery of extended functional loci. The input genomic DNA quantity emerges as the primary determinant of sequencing success. Samples with high DNA yields provide representative amounts of contigs coming confirmedly (matching sequences in the NCBI nucleotide database) or potentially (no-hit fraction) from myxomycetes, regardless of specimen age. In addition to target DNA, we revealed distinct signals of both anthropogenic contamination (human DNA and skin microflora) and natural substrate inhabitants, including oribatid mites and bacteria from dead wood, soil, and grass litter. Thus, even in old collections, metagenomic data still carry information regarding the substrate upon which the myxomycete developed. The results demonstrate that short-read genome skimming may help to integrate historical type material of myxomycetes into contemporary phylogenetic research. This method overcomes the length-dependent limitations of traditional Sanger sequencing, thus providing a roadmap for the future of museomics in myxomycetology.

## Introduction

Nomenclatural types serve as the foundation of biological systematics: every scientific name is ultimately anchored to a specific physical specimen preserved in a collection (Turland et al. 2018). Many discussions regarding diagnostic features, intraspecific variability, taxonomic boundaries, and phylogenetic relationships among species can potentially be resolved by reference to the type. The splitting of a single polymorphic species into several new ones must also rely on the type specimen; otherwise, it remains unclear which of the new species should retain the historical name (Winston 1999; Singh 2019). The loss or destruction of a type creates obstacles for any taxonomic revision, engendering nomenclatural uncertainty and forcing authors to interpret names arbitrarily.

With taxonomy entering the molecular era, DNA barcoding has become a major tool for species identification and delimitation. However, alongside new opportunities, this methodology has revealed a serious limitation: type specimens collected in the distant past cannot be analysed using routine methods applied to fresh material. Specifically, for *Myxomycetes*—a group of terrestrial protists with collectible fungus-like fructifications (Stephenson et al. 2008), the conventional age threshold for Sanger sequencing-based barcoding is typically estimated at 10–15 (20) years (Leontyev et al. 2015, 2026; Schnittler et al. 2017, 2020; Janik et al. 2020). This problem creates a fundamental gap between historical names, linked to non-barcoded types, and molecular data from contemporary collections (Leontyev et al. 2019a; Ronikier et al. 2022; Prikhodko et al. 2023). Without matching to type material, large datasets of thoroughly studied contemporary specimens cannot be reliably identified. This situation presents a major challenge, as approximately 85% of known myxomycete species were described before 2000 (Schnittler et al. 2025), leaving their type specimens unbarcodable by standard methods.

However, advances in paleogenetics have demonstrated that DNA is a relatively stable molecule, capable of persisting for hundreds of thousands of years (Pääbo 1989; Kjær et al. 2022). In this context, the rapid decline in sequencing success observed for myxomycetes appears paradoxical. The explanation lies in the fact that the obstacle to barcoding old collections is not the total destruction of DNA, but its fragmentation following cell death (Pääbo et al. 2004; Burrell et al. 2015; Brewer et al. 2019). If the genome is fragmented into segments shorter than the target marker locus, its PCR amplification coupled with classical Sanger sequencing becomes technically impossible, even if the specimen still contains a significant amount of DNA (Bakker and Hemerik 2023). Based on this rationale, many authors have already turned to next-generation sequencing methods, specifically the Illumina platform, which produces millions to billions of short nucleotide sequence reads. The results have been impressive: it has been shown that even a specimen age of 150–200 years is not a limit for successful molecular barcoding of plants and fungi stored in herbaria (Bakker 2017; Shumskaya et al. 2023). Natural history collections have proven to be a “repository of dead DNA”, a vast archive of genomic data that can be used for taxonomic, population, evolutionary, and ecological studies (Burrell et al. 2015; Bieker and Martin 2018). Consequently, the concept of museomics has emerged, encompassing the analysis of nucleic acid and protein molecules in museum collections (Guschanski et al. 2013; Raxworthy and Smith 2021).

Probably the most relevant and cost-effective approach for working with old specimens is genome skimming. This method is based on low-coverage random sequencing of total DNA, aiming not for full nuclear genome reconstruction, but rather for “skimming off” high-copy regions such as ribosomal and mitochondrial markers. Due to their multi-copy nature, these markers are represented in libraries significantly more frequently than single-copy nuclear genes, allowing for reliable molecular barcoding even with extremely low DNA yields (Straub et al. 2012; Zeng et al. 2018).

In the present study, we attempted to evaluate the feasibility of barcoding myxomycete herbarium collections ranging from 29 to 91 years in age using Illumina-based genome skimming. We aimed to test the effectiveness of this method for obtaining reliable genetic data from specimens with varying degrees of preservation and diverse DNA concentrations.

## Methods

### Material selection

The study material consisted of seven myxomycete specimens from various taxonomic groups, collected by R. Hagelstein, T.E. Brooks, I. Ammirati, R. McHugh, S.L. Stephenson, Y.K. Novozhilov, and M. Schnittler (see Table 1) and currently housed in the herbarium of the Institute of Botany and Landscape Ecology at the University of Greifswald, Germany (Suppl. material 1: Sheet A). The specimens were selected to represent an age gradient, with collection dates ranging from 1935 to 1997. Initially, eight specimens were selected; however, one was found to be completely devoid of DNA for unclear reasons and was excluded from the study. Our analysis of this particular specimen is detailed in a separate publication (Leontyev and Schnittler 2025). The selected specimens differed significantly in size and number of fruiting bodies, but all contained friable, uniformly coloured spore masses and lacked visible contamination by fungi or arthropods. Around 1 mm^3^ of spores were sampled from each specimen; the total spore count was not normalized.

**Table 1.** Sequencing data for the herbarium collections used in this study.

**Species**	***Fuligo cinerea***	***Trichia scabra***	***Lindbladia tubulina***	***Cribraria mirabilis***	***Lycogala epidendrum***	***Lindbladia tubulina***	***Lycogala epidendrum***
Species ID	sc11445	sc11426	sc12075	sc10428	UARK25089	sc2833	sc11299
Age of collection, years	91	64	51	47	41	33	29
DNA concentration before WGA, ng/μL	0.55	4.53	2.61	1.85	1.73	8.57	4.24
DNA concentration after WGA, ng/μL	5.07	–	9.14	4.51	2.76	–	4.94
DNA amount used for sequencing, ng	365	158	658	325	199	300	356
Total sequence length, Mbp	1532.1	1670.7	1133.9	1463.4	1425.4	2353.5	1516.7
Total assembly length, Mbp	11.1	162	9.1	2.6	1.7	84.1	16.8
No-hit fraction length, Mbp	5.1	108	4.7	2	1.4	82.3	16.6
*Eumycetozoa* fraction length, kbp	1.5	49.7	0.4	0.2	0.5	111	5.1
*Eumycetozoa* contigs count	6	17	2	1	2	19	11
No-hit & *Eumycetozoa* N50, bp	251	266	214	207	208	4190	243
No-hit & *Eumycetozoa* longest contig, kbp	8.2	41.6	4.1	1.81	1.63	114	4.7
No-hit & *Eumycetozoa* mean GC content, %	50.5	53.7	35.7	37.1	32.8	37.9	35.7
Interpretation of dominant contaminants	Decomposition microbiota: Saprophytic bacteria typical of long-term decay.	Forest substrate: Decaying wood microflora and photosynthetic cyanobacteria.	Mixed noise: indoor dust, mammalian DNA, and forest mesofauna (mites).	Indoor dust: fungal and bacterial microflora of residential areas.	Indoor dust: skin microflora and associated dermatophytic fungi.	Soil microbiota: predominantly nitrogen-fixing soil bacteria.	Anthropogenic noise: trace human DNA and typical skin microflora.

### DNA library preparation and sequencing

Following the recommendations of Shumskaya et al. (2023), we extracted DNA from the specimens using the filter-based QIAamp DNA Micro Kit (Qiagen, Germany), following the “Isolation of Genomic DNA from Tissues” protocol supplied by the manufacturer. A key feature of this kit is the presence of carrier RNA, i.e. polyA molecules that non-specifically bind short DNA fragments, preventing them from passing through the filter pores. For each sample, 50 µL of DNA solution were obtained. The concentration of the resulting product was measured using a DeNovix DS-11 FX fluorometer (DeNovix Inc. USA) following the TN 143 Broad Range Assay Detailed Protocol (DeNovix Inc. USA). After concentration measurement, the samples were divided in two groups. The first group, where the extracted DNA amount reached 150 ng (sc11426 and sc2833), was directly used for sequencing. For the second group (the rest of the samples, with the lower amounts of DNA), a 2.5 µL aliquot was taken for whole-genome amplification (WGA) using the 4BB TruePrime WGA Kit (4basebio, UK). The amplification process was conducted for 6 hours. Then, 35 µL of genomic DNA solution (first group) or 72 µL of WGA products (second group) with final amount of DNA ranging from 158 to 658 ng (see Table 1), were sent to Eurofins Genomics (Germany) for genome skimming on the Illumina NovaSeq (USA) platform, using the WGS Bacteria/Yeast/Fungi protocol with a sequencing depth of 5 million read pairs (2x 150 bp) per sample.

### Bioinformatic processing

The demultiplexed, adapter-trimmed paired-end Illumina sequencing data were screened for quality and presence of remaining adapter sequences using FastQC v0.11.9 (Andrews 2010). To obtain a high sensitivity of detection of low-abundance genomic signal expected from old DNA samples, the reads were assembled *de novo* using a metagenomic assembler metaSPAdes v4.0.0 (Nurk et al. 2017), which is equivalent to running SPAdes with the “--meta” flag. No prior quality-based read trimming or filtering was done before the assembly. The assemblies were run on the High-Performance Computing cluster of the University of Greifswald. For the sample *Lindbladia tubulina* sc2833, which had the highest input DNA amount and the largest sequencing data volume, this assembly method failed with an out-of-memory error. Therefore, the assembly for this sample was made using the standard assembly mode of SPAdes v4.0.0 (Prjibelski et al. 2020).

For taxonomic profiling of contigs, each resulting assembly was searched against a locally installed NCBI nucleotide database (“core_nt”, updated 31 Jan 2026) using BLASTn v. 2.5.0+ in megablast mode (Camacho et al. 2009). BLAST output was generated in tabular format (“outfmt 6”) including subject TaxIDs, and these TaxIDs were linked to the NCBI taxonomy using the NCBI “taxdump” files.

To estimate per-contig sequencing depth, the original reads of each sample were mapped back to the corresponding assembly using strobealign v. 0.17.0 (Sahlin 2022). Alignment output (SAM, Sequence Alignment Map) was converted to sorted and indexed BAM (Binary Alignment Map) files using samtools v. 1.12 (Danecek et al. 2021). Taxonomic assignments, GC content, contig length, and read depth were integrated and visualized with BlobToolKit v. 4.5.0 (Challis et al. 2020). For each sample, BlobTools2 was used to create a project from the assembly FASTA, BLAST hits, and BAM coverage file, and summary snail and blob plots were produced to assess the distribution of contigs and read support across inferred taxonomic groups at different taxonomic levels.

For every contig where the BLAST search over a local core_nt database found a match to an organism belonging to *Eumycetozoa*, we additionally conducted a thorough online BLAST search over the NCBI GenBank Nucleotide database (see Suppl. material 1: Sheet B for a list of the best 10 matches with the smallest e-values and Sheet C for a list of genes found).

## Results

### Identification of the target genome fragments

For all seven samples, we obtained a substantial volume of raw genetic data. The total sequence length was relatively uniform, ranging from 1134 to 2354 Mbp (Table 1). However, the total length of the assembled contigs varied by two orders of magnitude, ranging from 1.7 Mbp in *L. epidendrum* sc25089 to 162.0 Mbp in *T. scabra* sc11426. Compared to the genome size of *Badhamia polycephala* (Miller et al. 2021), this corresponds to an estimated genome coverage between 0.7% and 65%.

**Figure 1.**
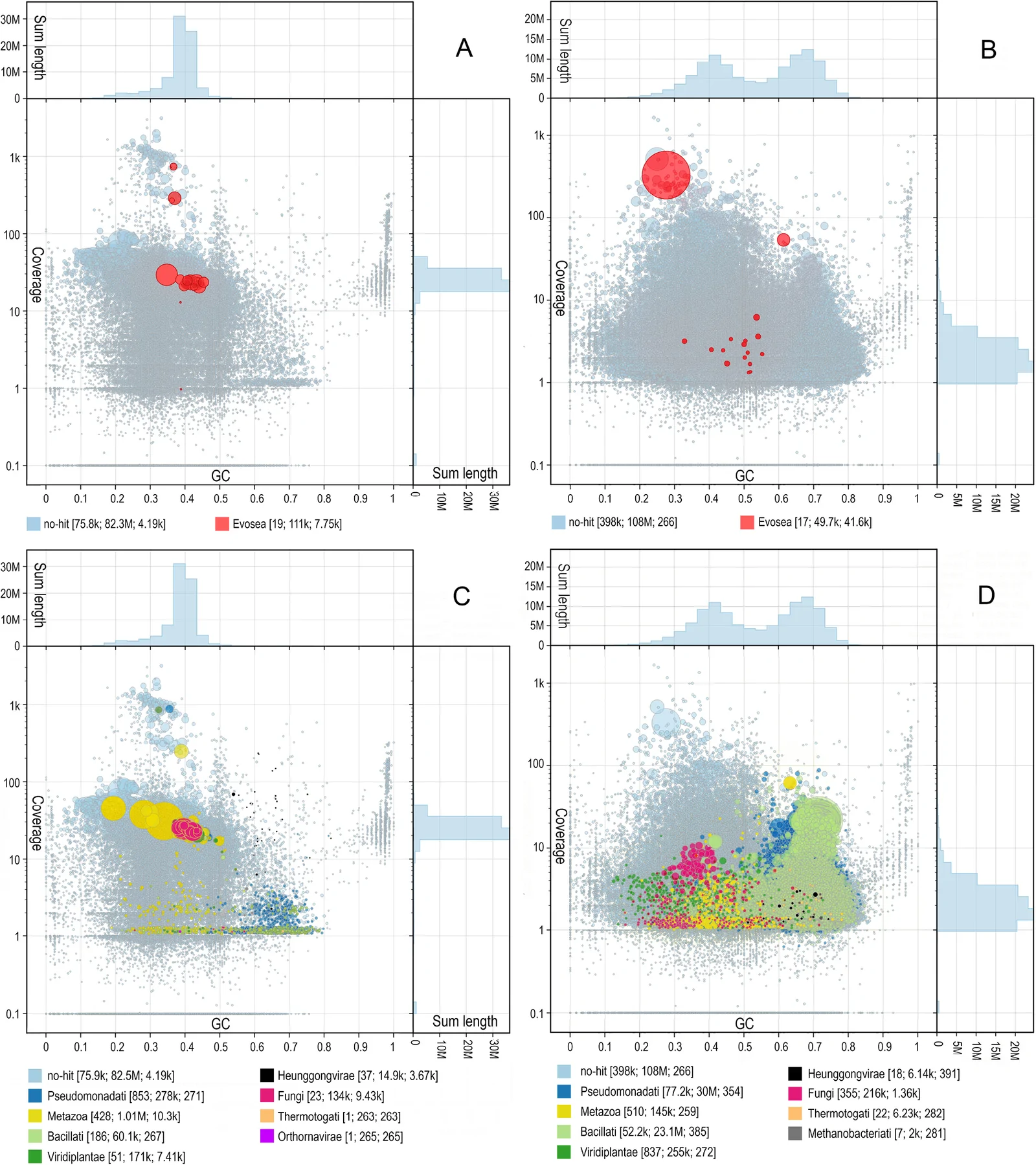
Blob plot summary for assembled contigs of *Lindbladia tubulina* sc2833, 33 years old (**A, C**) and *Trichia scabra* sc11426, 64 years old (**B, D**). Axes: base coverage (logarithmic) against GC proportion. Circles are sized in proportion to contig length. Histograms show the distribution of contig length sum along each axis. **A, B**. *Eumycetozoa* contigs shown against no-hit fraction. **C, D**. Leading groups of the sample metagenome at the level of kingdoms are shown.

Visualization of the assemblies via blob plots (https://doi.org/10.5281/zenodo.20467237) demonstrates significant differences in the data structure between samples. In each specimen, there were a number of contigs (1–19) unambiguously classified as belonging to *Eumycetozoa* based on BLAST hits (Fig. 1, Tables 1, 2). However, most sequences within the resulting assemblies belonged to the no-hit fraction—sequences with no matches in the NCBI database. We hypothesize (see Discussion) that a substantial proportion of this fraction is composed of myxomycete genetic material, which remains unidentified because genomic data for myxomycetes are still extremely sparse (Takano et al. 2001; Schaap et al. 2016; Kang et al. 2017). In the sample *Lindbladia tubulina* sc2833 (Fig. 1A, C), the no-hit fraction accounted for 82.3 Mbp and had a unimodal GC content distribution with highest contig density within a 35% to 45% range and a predominant base coverage of 20–40×, matching those of the contigs identified as fragments of myxomycete nuclear genome. In *Lycogala epidendrum* UARK25089 (https://doi.org/10.5281/zenodo.20467237), the no-hit fraction was smaller (1.4 Mbp) but maintained a characteristic distribution profile, allowing its identification as a residual signal of the nuclear genome. In *Trichia scabra* sc11426 (Fig. 1B, D), the no-hit contig fraction had a bimodal GC content distribution, with the second peak at around 68%, suggesting heavy contamination by prokaryotic DNA. In samples with low input DNA amount and high fragmentation, such as *Cribraria mirabilis* sc10428 and *Fuligo cinerea* sc11445 (https://doi.org/10.5281/zenodo.20467237), the data were represented by a sparse cloud of short contigs with a broad unimodal GC content distribution and the base coverage peaking around one.

**Table 2.** Molecular identification of old herbarium collections used in the study: a – sequence fragment length (bp); b – base coverage of the fragment (number of reads); c – match according to Nucleotide BLAST (%).

**Specimen ID**	**Identified taxon (ribogroup)**	**nucSSU**			**mtSSU**			**COI**			**EF1a**		
		**a**	**b**	**c**	**a**	**b**	**c**	**a**	**b**	**c**	**a**	**b**	**c**
sc11445	*Fuligo cinerea*	157	4	100.0	–	–	–	–	–	–	–	–	–
sc11426	*Trichia scabra*	2952	32	99.8	–	–	–	41607	197	100.0	507	2	87.7
		–	–	–	–	–	–	–	–	–	239	1	99.6
		–	–	–	–	–	–	–	–	–	226	1	87.9
sc12075	*Lindbladia tubulina*	169	1	92.7	–	–	–	–	–	–	259	1	88.2
sc10428	*Cribraria mirabilis*	220	1	93.0	–	–	–	–	–	–	–	–	–
UARK25089	*Lycogala epidendrum*	318	1	99.7	–	–	–	145	1506	100.0	–	–	–
sc2833	*Lindbladia tubulina*	7755	127	100.0	2310	330	100.0	–	–	–	10830	10	91.9
		2106	8	100.0	–	–	–	–	–	–	4847	8	86.4
		1121	127	100.0	–	–	–	–	–	–	–	–	–
sc11299	*Lycogala epidendrum*	1129	4	99.4	620	13	99.0	878	34	81.6	233	1	89.0
		696	3	94.0	–	–	–	223	14	94.6	–	–	–
		381	3	87.4	–	–	–	142	46	92.9	–	–	–
		161	151	100.0	–	–	–	–	–	–	–	–	–

**Figure 2.**
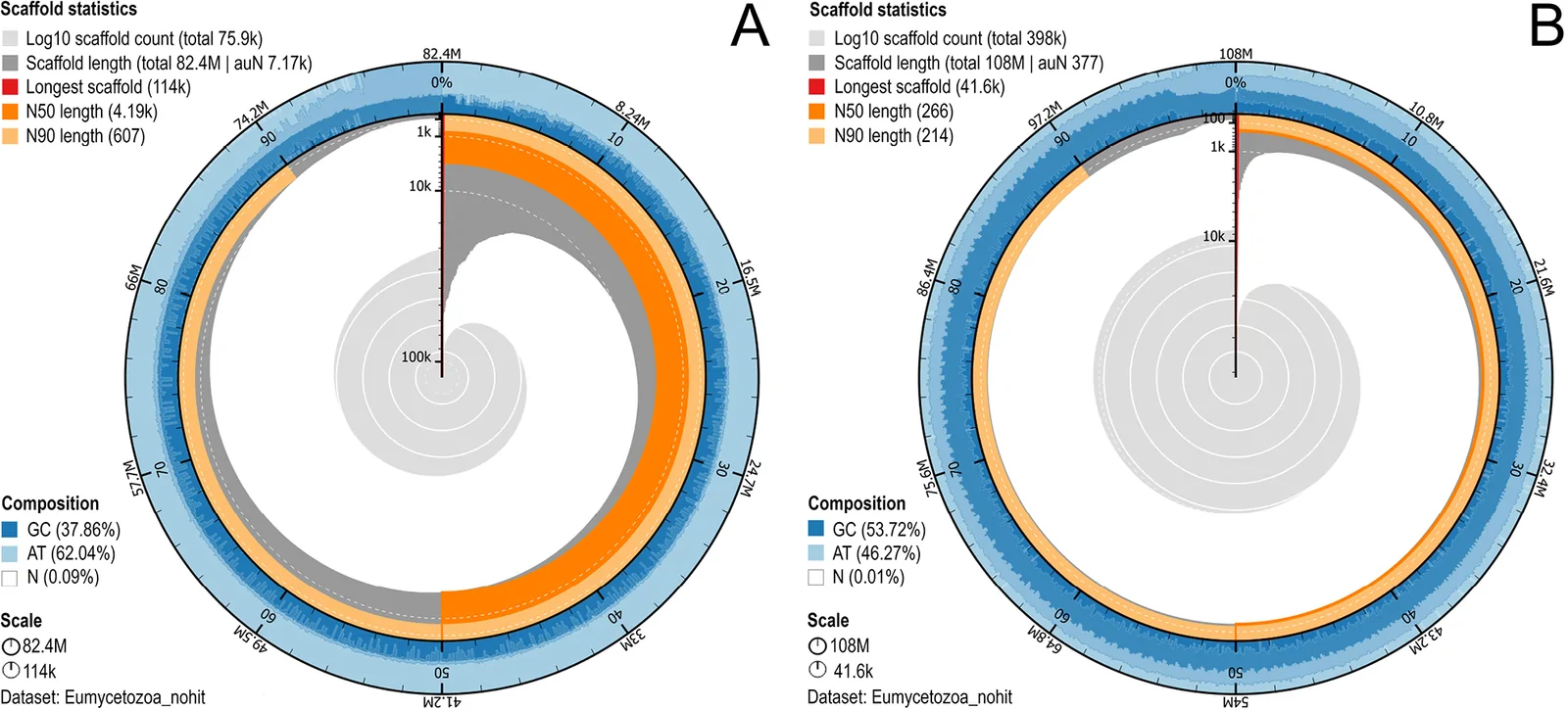
Snail plot of assembly statistics for *Lindbladia tubulina* sc2833, 33 years old (**A**), and *Trichia scabra* sc11426, 64 years old (**B**). The dark grey area represents the sequence length distribution, with the radius scaled to the longest sequence (red line, 105k and 13.2k, respectively). Orange and pale-orange arcs indicate N50 and N90 lengths, respectively. The pale grey spiral shows the cumulative sequence count on a logarithmic scale (white lines show orders of magnitude). The outer bands visualize GC (blue), AT (pale-blue), and non-identified bases (white) content distributions, binned consistently with the inner sequence data.

In the combined no-hit & *Eumycetozoa* fraction of all samples except *L. tubulina* sc2833, the N50 values vary between 207–266 bp (Table 1, Fig. 2) and the statistical mode of read length distribution varies between 56 and 237 bp. This reflects the dominance of short unresolved fragments in the assembly graph that likely resulted from dead ends, short sequence repeats, rare variants, abundant contaminants, low-coverage sequences or sequencing errors. These short and low-coverage contigs are not filtered out from the results by metaSPAdes. The sample *L. tubulina* sc2833 stands out, with the N50 reaching 4.19 Kbp, which is likely explained by the higher input DNA amount and the different assembly mode applied to it (standard SPAdes mode – see Methods).

**Figure 3.**
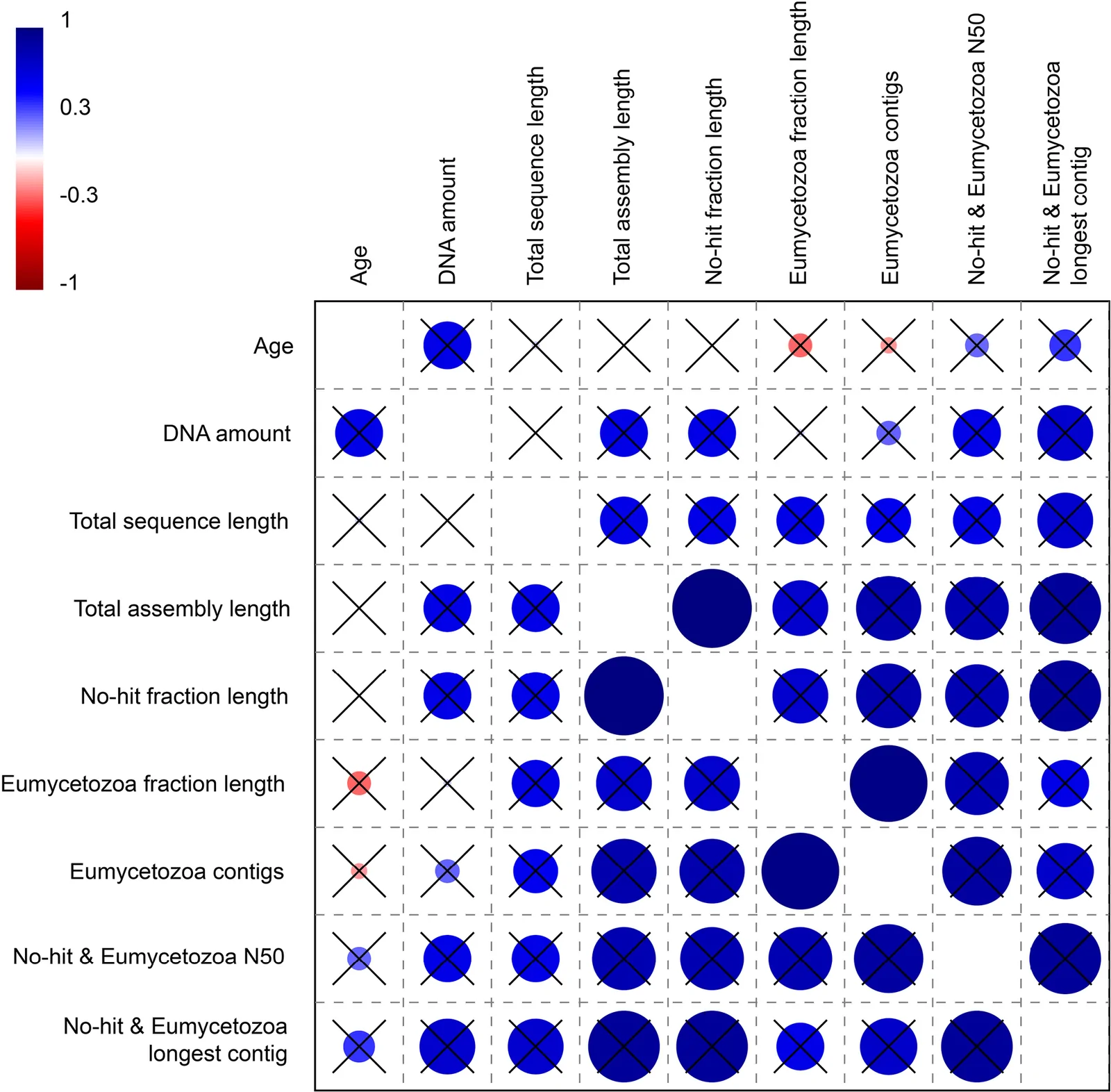
Spearman correlation matrix (*r_s_*) between specimen age, DNA concentration, and bioinformatic characteristics of the sequencing data, calculated for five specimens sequenced after WGA. Blue and red colours represent a positive and negative correlation respectively. The size of the circles is proportional to the absolute value of the correlation coefficient *r_s_*. Non-significant correlations (*p* > 0.05) are marked with crosses. If the Bonferroni correction is applied, all values are insignificant. The diagram was generated using the PAST software.

Within the examined set of samples, specimen age did not show a statistically significant linear correlation with genomic data quality. The two specimens yielding the highest amounts of DNA (*L. tubulina* sc2833 and *T. scabra* sc11426) span widely different ages (33 and 64 years, respectively) and are not the youngest among the studied collections (Table 2). Similarly, no significant correlation was detected between specimen age and DNA yield, read length, or the number of recovered *Eumycetozoa* contigs across the WGA-treated subset (Fig. 3). While DNA degradation over time is indisputable, these observations suggest that within a timeframe of several decades, initial preservation conditions and sample-specific properties may outweigh chronological age as a limiting factor.

### DNA barcodes in genomic assemblies

Although the detection of *Eumycetozoa* within the resulting data remains challenging due to the lack of reference genomes, we succeeded in identifying anchor contigs for all seven specimens that correspond to their preliminary morphological identification (Suppl. material 2). Despite the paucity of these fragments, their presence is critical for specimen barcoding—the primary objective of genome skimming for old herbarium collections. It is important to emphasize that for most specimens the identified *Eumycetozoa* markers are localized within the main no-hit cloud (Fig. 1; https://doi.org/10.5281/zenodo.20467237). The Sheet B in the Suppl. material 1 lists the ten best BLAST hits for each contig and the detected genes.

Nuclear small subunit ribosomal gene (nucSSU) sequences were identified for all specimens. Their length varied from 157 to 7755 bp, and base coverage ranged from 1 to 127 copies (Table 2). For six out of seven specimens, the detected fragments of nucSSU at least partially covered the ca. 600 bp fragment (Suppl. material 3) conventionally used for DNA barcoding in myxomycetes (Leontyev and Schnittler 2017).

The total number of recognized genes reached 28 (Suppl. material 1: Sheet C). Among them, the eukaryotic elongation factor 1-alpha (EF1a) gene was detected in four specimens, with the contig length exceeding 10 kbp in *L. tubulina* sc2833. For three specimens, contigs containing the mitochondrial cytochrome c oxidase subunit I (COI) gene were obtained, varying in length (up to 41 kb) and base coverage (14–1506). Sequences including the mitochondrial small subunit ribosomal gene (mtSSU) were recovered for only two specimens, which also differed in length (up to 2310 bp) and base coverage (13–330). Other identified genes like actin, alpha-tubulin, and several tRNAs have not been studied for bright-spored myxomycetes; consequently, the matches found did not allow for species-level identification, as the closest analogues were dark-spored myxomycetes and dictyostelids (Suppl. material 1: Sheet B).

### Analysis of contamination and associated biota

Taxonomic interpretation of non-target contigs revealed a complex metagenomic background, the composition of which varies depending on specimen age, substrate conditions, and archival history (https://doi.org/10.5281/zenodo.20467237). The anthropogenic fraction, primarily represented by human DNA, was recorded in all examined specimens. The proportion of this fraction correlates with the total DNA yield: in libraries with low target material content (*C. mirabilis* sc10428, *L. epidendrum* sc25089), anthropogenic noise becomes dominant. In close association with human DNA, a skin microflora complex was identified, including the bacterium *Cutibacterium acnes*, representatives of the genus *Staphylococcus*, and lipophilic yeasts of the genus *Malassezia*. Domestic cattle DNA (*Bos taurus*) found in several samples likely also belongs to the anthropogenic fraction.

The second metagenomic fraction is conditionally natural, reflecting the substrate inhabitants and ecosystem relationships of the myxomycetes. Arthropod DNA was detected selectively; the most pronounced signal was recorded in specimen *L. tubulina* sc12075, where oribatid mites of the genus *Oppiella*, common inhabitants of decaying wood (Maraun and Scheu 2000), were identified. The bacterial component of this fraction is represented by organisms characteristic of forest ecosystems: the cyanobacterium *Nostoc* and the betaproteobacterium *Variovorax* (associates of rotting wood) were noted in specimen *T. scabra* sc11426, while nitrogen-fixing soil bacteria of the genus *Bradyrhizobium* dominate in *L. tubulina* sc2833. In the oldest specimen of the series, *F. cinerea* sc11445, a predominance of putrefactive bacteria of the genus *Bacillus* was observed. The archaeal component is limited to nitrifying soil forms (*Nitrososphaera*). The viral fraction is represented by fragments of bacteriophages.

## Discussion

The obtained data demonstrate that genome skimming is an effective method for the molecular identification of myxomycete herbarium specimens, even when the material is of significant age. We successfully extracted at least one of the four markers we searched for (nucSSU, mtSSU, EF1a, COI) from all specimens, including the oldest collection, *Fuligo cinerea* (1935). For six out of the seven specimens, more than one marker was identified.

The lack of a direct correlation between specimen age and the quality of the resulting data should not be interpreted as an absence of time-dependent DNA degradation. However, it leads to an important conclusion: within the studied timeframe, the informativeness of the assembly does not critically deteriorate solely as a function of chronological age. This suggests that even a specimen age exceeding 100 years is not necessarily an obstacle to genome skimming, as previously demonstrated for vascular plants and fungi (Bieker and Martin 2018; Shumskaya et al. 2023). At the same time, the starting DNA amount is crucial for obtaining high-quality data. Large specimens with a high quantity of spores yielded good results regardless of their age.

The dominance of the no-hit fraction in all assemblies is likely explained by the critical lack of genomic data for myxomycetes, particularly for the bright-spored group, *Lucisporomycetidae* (Leontyev et al. 2019b), represented in our study by all specimens except for *F. cinerea*. The correspondence of the GC content and coverage profile of the no-hit fraction with the identified anchor nucSSU contigs allows us to preliminarily consider this fraction as partially belonging to the nuclear genome of the target organism. The consistency of the GC content within the no-hit fractions provides some support for this assumption: since GC content is a group-specific genomic feature (Hildebrand et al. 2010), the observed clustering suggests a single taxonomic source. However, this assumption requires further evidence. Given the confounding effects of low coverage, short contig lengths, and potential background noise, we emphasize that the no-hit fraction cannot be assumed to entirely represent the target myxomycete DNA. Moreover, even sequences identified by BLAST as belonging to *Eumycetozoa* may represent the contamination by other myxomycete species. This methodological problem can only be resolved by creating high-quality *de novo* reference genomes using Hi-Fi PacBio and Hi-C technologies for all key groups of myxomycetes. Without such a roadmap, the potential of genome skimming for archival collections will remain limited to a narrow set of standard barcodes.

A considerable challenge is the high contamination load, which creates noise within the genomic data. Notably, specimens subjected to WGA generally exhibited higher contamination levels and lower yield of target DNA. This issue may be a direct consequence of the WGA process, which preferentially amplifies younger and less-degraded DNA of anthropogenic contaminants rather than the target DNA. Unfortunately, our data are insufficient to fully evaluate the impact of this factor, as the two specimens that did not undergo WGA and yielded more target DNA initially possessed the highest DNA concentrations. Consequently, the suitability of WGA for genome skimming remains an open question.

Genome skimming has provided insights not only into the target species but also into the ecological context of the specimen. The detection of mite DNA, lignotrophic betaproteobacteria (*Variovorax*), and cyanobacteria (*Nostoc*) allows for a partial reconstruction of the decaying wood communities at the time of collection. The components of the soil microbiome, such as nitrogen-fixing bacteria (*Bradyrhizobium*) and nitrifying archaea (*Nitrososphaera*), may as well represent the same ecosystem. A particularly intriguing case is the predominance of *Bacillus* spp. in the specimen *F. cinerea* sc11445. This myxomycete typically develops on dead herbaceous substrates, such as rotting straw, where mass development of *Bacillus* spp. is frequently observed (Mandic-Mulec et al. 2015). Thus, even in a 90-year-old specimen, metagenomic data still carry information regarding the substrate upon which the myxomycete developed. Undoubtedly, a similar study of fresh material could provide a wealth of data on the topical and trophic relationships of myxomycetes, including the composition of the microorganisms on which their plasmodia feed.

Even in the absence of comprehensive, annotated reference genomes for all myxomycete taxonomic groups, the approach demonstrated here still offers an effective solution for resolving taxonomic uncertainties. Recent studies confirmed the effectiveness of strategies based on genetic distance (Borg Dahl et al. 2018; Yatsiuk et al. 2025), branch length (Leontyev et al. 2026), and recombination (Shchepin et al. 2022) for reliable species delimitation in myxomycetes. Therefore, in cases where a nominal species, such as *Reticularia splendens*, *Trichia varia* or *Tubifera dimorphotheca* (Feng and Schnittler 2015; Leontyev et al. 2025, 2026), comprises a complex of a few semi-cryptic biological species, obtaining one or two species-specific barcoding fragments from the type collection could be sufficient to determine which biological species the type belongs to. If a type yields only highly fragmented, low-quantity DNA, the following approach can be employed. By sequencing one modern voucher for each recognized biospecies, researchers can establish a reference set for the barcoding of old collections. The Illumina-based reference library will provide the necessary data to accurately assign the type to its corresponding lineage.

NGS is not the only alternative for specimen barcoding of old collections with highly fragmented DNA. An alternative approach could be the design of primers specifically for the amplification of short barcode fragments that fall within the length range of old, degraded DNA. The authors have achieved success through this method and will report on it in a follow-up publication.

## Conclusion

The proposed method opens new prospects for the revision of herbarium collections and the construction of a molecular bridge between historical types and modern species concepts. The ability to extract genomic information from almost century-old specimens allows for the integration of myxomycete nomenclatural types, facilitating the entry of myxomycetological research into the era of phylogenomics.

## Additional information

### Artificial Intelligence (AI) use

The authors accept full responsibility for the content of the manuscript, including the disclosure of any use of AI.

No AI tools were used in the preparation of this manuscript.

### Adherence to national and international regulations

All the fungal strains used in this study have been legally obtained, respecting the Convention on Biological Diversity (Rio Convention).

### Author ORCIDs

D.V. Leontyev https://orcid.org/0000-0002-4122-1091

M. Schnittler https://orcid.org/0000-0003-0909-5627

O.N. Shchepin https://orcid.org/0000-0001-9327-7655

### Data availability

All of the data that support the findings of this study are available in the main text or Supplementary materials. In addition, complete sets of genomic blob-plots and snail-plots for all studied specimens are deposited in Zenodo repository at https://doi.org/10.5281/zenodo.20467237).

## Supplementary materials

Supplementary material 1 Supplementary tables Data type xlsx Explanation note Sheet A. Collection data for the specimens used in the study. Sheet B. MultiBLAST for contigs where myxomycete genes could be identified. The established DNA barcode (nucSSU / 18S rRNA gene) is shown in red. Sheet C. Genes recognised in contigs identified as *Eumycetozoa*. Standard barcoding and phylogenetic markers are shown in red. The total length / base coverage are shown for each contig (xlsx file). https://binary.pensoft.net/file/1764441This dataset is made available under the Open Database License (http://opendatacommons.org/licenses/odbl/1.0/). The Open Database License (ODbL) is a license agreement intended to allow users to freely share, modify, and use this Dataset while maintaining this same freedom for others, provided that the original source and author(s) are credited. Dmytro V. Leontyev, Martin Schnittler, Oleg N. Shchepin

Supplementary material 2 Sequences obtained from each studied specimen Data type zip Explanation note Sequences obtained from each studied specimen by Illumina NovaSeq-based genome skimming and recognised as *Eumycetozoa* (ZIP archive of FASTA files). https://binary.pensoft.net/file/1764442This dataset is made available under the Open Database License (http://opendatacommons.org/licenses/odbl/1.0/). The Open Database License (ODbL) is a license agreement intended to allow users to freely share, modify, and use this Dataset while maintaining this same freedom for others, provided that the original source and author(s) are credited. Dmytro V. Leontyev, Martin Schnittler, Oleg N. Shchepin

Supplementary material 3 Alignment of the fragment of nucSSU Data type fas Explanation note Alignment of the fragment of nucSSU (18S rRNA gene) used for species barcoding in our data and reference barcodes of the respective taxa obtained by Sanger sequencing (FASTA file). https://binary.pensoft.net/file/1764443This dataset is made available under the Open Database License (http://opendatacommons.org/licenses/odbl/1.0/). The Open Database License (ODbL) is a license agreement intended to allow users to freely share, modify, and use this Dataset while maintaining this same freedom for others, provided that the original source and author(s) are credited. Dmytro V. Leontyev, Martin Schnittler, Oleg N. Shchepin
